# Association between serum calcium levels and prognosis, hematoma volume, and onset of cerebral hemorrhage in patients undergoing hemodialysis

**DOI:** 10.1186/s12882-019-1400-4

**Published:** 2019-06-07

**Authors:** Mineaki Kitamura, Yohei Tateishi, Shuntaro Sato, Satoko Kitamura, Yuki Ota, Kumiko Muta, Hiroshi Yamashita, Tadashi Uramatsu, Yoko Obata, Yasushi Mochizuki, Masaharu Nishikido, Tsuyoshi Izumo, Takashi Harada, Satoshi Funakoshi, Takayuki Matsuo, Akira Tsujino, Hideki Sakai, Hiroshi Mukae, Tomoya Nishino

**Affiliations:** 1 Division of Blood Purification, Nagasaki University Hospital, Nagasaki, Japan; 2 Department of Nephrology, Nagasaki University Hospital, Nagasaki, Japan; 3 Department of Neurology and Strokology, Nagasaki University Hospital, Nagasaki, Japan; 4 Clinical Research Center, Nagasaki University Hospital, Nagasaki, Japan; 5 Department of Urology, Nagasaki Medical Center, Omura, Japan; 6 Department of Neurosurgery, Nagasaki University Graduate School of Biomedical Sciences, Nagasaki, Japan; 7 Department of Nephrology, Nagasaki Renal Center, Nagasaki, Japan; 8 Department of Respiratory Medicine, Unit of Basic Medical Sciences, Nagasaki University Graduate School of Biomedical Sciences, Nagasaki, Japan

**Keywords:** Cerebral hemorrhage, Hemodialysis, Serum calcium

## Abstract

**Background:**

High serum calcium levels should be avoided in patients on hemodialysis (HD) because they can induce cardiovascular diseases and worsen the patient’s prognosis. In contrast, low serum calcium levels worsen the prognosis of patients with cerebral hemorrhage in the general population. So far, whether serum calcium levels in patients on HD are associated with cerebral hemorrhage remains unknown. This study aimed to reveal the association between serum calcium and cerebral hemorrhage in patients on HD, including in-hospital death, volume of hematoma, and onset of cerebral hemorrhage.

**Methods:**

This cross-sectional case-control study included 99 patients on HD with cerebral hemorrhage at a single center between July 1, 2007 and December 31, 2017. Controls included 339 patients on HD at a single HD center between July 1, 2011 and June 30, 2012. Data on serum calcium level, patient demographics, and comorbid conditions were collected, and associations between cerebral hemorrhage and subsequent death were evaluated by multivariate logistic regression analysis. Further, the association of these backgrounds and hematoma volume was evaluated by multiple regression analysis.

**Results:**

Of the 99 patients, 32 (32%) died from cerebral hemorrhage. The corrected serum calcium level (odds ratio [OR], 2.49; 95% confidence interval [CI], 1.43–4.35; *P* < 0.001) and antiplatelet drug use (OR, 3.95; 95% CI, 1.50–10.4; *P* = 0.005) had significant effects on the prognosis. Moreover, the corrected serum calcium (*P* = 0.003) and antiplatelet drug use (*P* = 0.01) were significantly correlated with hematoma volume. In the patients, the corrected serum calcium level (OR, 1.54; 95% CI, 1.07–2.22; *P* = 0.02) was associated with the onset of cerebral hemorrhage, as was pre-hemodialysis systolic blood pressure (per 10 mmHg) (OR, 1.40; 95% CI, 1.23–1.59; P < 0.001).

**Conclusions:**

Although the precise mechanisms remain unknown, a high serum calcium level is associated with cerebral hemorrhage in patients on HD. Thus, we should pay attentions to a patient’s calcium level.

**Electronic supplementary material:**

The online version of this article (10.1186/s12882-019-1400-4) contains supplementary material, which is available to authorized users.

## Background

Stroke is a major cause of death and the leading cause of disability in patients on hemodialysis (HD) worldwide, where its incidence is several times higher than in the general population [1–6]. Although the recent incidence of cerebral infarction was found to be higher than that of cerebral hemorrhage in patients on HD [7], cerebral hemorrhage had a greater impact on patients’ prognoses compared with cerebral infarction [4, 6, 8]. To prevent cerebral hemorrhage or mitigate the severity of cerebral hemorrhage, modifiable factors in patients on HD are crucial.

Several traditional risk factors for cerebral hemorrhage in patients on HD have been elucidated, such as male sex [9], absence of antihypertensive drug [10], pre-dialysis hypertension [2, 3, 5, 7, 9], low KT/V [3], and high blood hemoglobin [11]. In addition to chronic kidney disease (CKD)-mineral bone disorder, a high serum phosphate level [9] and high intact parathyroid hormone (iPTH) level [12] are known risk factors for cerebral hemorrhage in patients on HD.

Since multiple studies have reported that a high calcium concentration is associated with cardiovascular diseases and a poor prognosis in patients with CKD [13–15], the KDIGO guidelines have changed the recommendations for avoiding hypercalcemia in patients on HD, although the treatment range of serum calcium has been left unchanged [16, 17].

In contrast, studies that were not restricted to patients with end-stage renal disease (ESRD) have demonstrated that a low serum calcium level worsens patients’ prognoses and enlarges the hematoma volume in acute cerebral hemorrhage [18–20].

We decided to investigate the contradicting content of whether serum calcium levels in HD patients with cerebral hemorrhage affect patients’ prognosis or onset. This study aimed to reveal the association between serum calcium and cerebral hemorrhage in patients on HD, including in-hospital death, volume of hematoma, and onset of cerebral hemorrhage.

## Methods

### Study design

This is a cross-sectional case-control study in the same medical zone. The cases were of patients on HD with cerebral hemorrhage who were admitted to Nagasaki University Hospital or of patients on HD who developed cerebral hemorrhage at Nagasaki University Hospital during their stay for other treatments, but patients with traumatic hemorrhage, subarachnoid hemorrhage, and hemorrhage after ischemic stroke were excluded. The study period was between July 1, 2007, and December 31, 2017. Patients were diagnosed with cerebral hemorrhage using computed tomography (CT) at Nagasaki University Hospital within 3 days of experiencing neurological symptoms. Cases of traumatic hemorrhage were excluded based on the decision of the neurosurgeons of our hospital and imaging diagnosis at admission. Treatment strategies were discussed by multiple neurosurgeons and neurologists. The controls included patients who underwent HD between July 1, 2011, and June 30, 2012, in Nagasaki Renal Center, which is the largest HD facility in Nagasaki City. Almost a quarter of patients on HD at Nagasaki City medical zone were enrolled. Patients who did not undergo HD in their birth month were excluded because data collected in the birth month and on birthdays were considered substituted events.

### Data collection

Baseline characteristics of patients were collected through a retrospective review of hospital medical records and medical referral letters. In addition, data on the HD status just before admission, activities of daily living (ADL) before onset, and medications as well as laboratory data obtained closest to the event were collected from the medical referral letters. The hematoma volume was determined from a CT scan on admission or just after onset by a simplified formula as follows: maximum transverse diameter × maximal anteroposterior diameter × maximal superoinferior diameter × 1/2 [3, 21], which was natural-log transformed as in a previous study [22]. The onset of time was estimated from medical referral letters; if it was impossible, we assigned noon as the time of onset, as in previous reports [23]. The Glasgow Coma Scale (GCS) score, National Institutes of Health Stroke Scale (NIHSS) score [24], and severity of cerebral hemorrhage were defined by ADL at discharge, which was evaluated by the modified Rankin scale (mRS) [25], as follows: 0, no symptoms at all; 1, no significant disability despite symptoms; 2, slight disability; 3, moderate disability (able to walk without assistance); 4, moderately severe disability; 5, severe disability (bed ridden); and 6, death.

For the controls (control group), data were collected from the medical records of Nagasaki Renal Center. Similar to that in patients with cerebral hemorrhage (cerebral hemorrhage group), data were collected in the period before the patient’s birthday. The corrected serum calcium levels were obtained using the Payne equation [26]. A ratio of 1:200 was applied to convert the darbepoetin-alfa and epoetin-beta pegol doses to their equivalent epoetin doses [27].

### Statistical analysis

Categorical variables were expressed as number (%), whereas continuous values were expressed as mean ± standard deviations (SD). Non-normally distributed data were presented as median values with interquartile ranges. Statistical analyses were performed using JMP 13 software (SAS Institute Inc., NC, USA). Wilcoxon rank sum test and the chi-squared test were used to evaluate differences between groups. Patients with cerebral hemorrhage were divided into four groups (Q1–Q4) with the cutoff values being the 25th, 50th, and 75th percentiles of the corrected serum calcium levels. Analysis of variance and Cochran-Armitage test were used to demonstrate the trend. Univariate and multivariate logistic regression analyses were also performed.

In the multivariate logistic regression analyses for the prognosis of cerebral hemorrhage patients, model 1 was only adjusted for age and sex, and parameters of model 2 were determined using a stepwise model (mixed method) with an inclusion criterion of *P* < 0.1 in univariate logistic regression analysis in addition to the corrected serum calcium level. In the comparison of the cerebral hemorrhage group and control group, model 1 adopted the risk factors of traditional cerebral hemorrhage, including dialysis vintage, history of diabetes, systolic blood pressure at initiation of HD proximal to onset, hemoglobin, phosphate, iPTH, and corrected serum-calcium. In model 2, parameters were determined using a stepwise model (mixed method) as described above.

Multiple regression analysis was performed to predict hematoma volume. The constitutional parameters were age, sex, and those associated with hematoma volume, such as systolic blood pressure (sBP) pre-HD [3] and corrected serum calcium levels [18, 20], and residual parameters were selected using the stepwise method (mixed method). An inclusion criterion of P < 0.1 in the univariate logistic regression analysis was used. Adjusted odds ratios (ORs) and 95% confidence intervals (CIs) were calculated. A *P* value < 0.05 was considered statistically significant.

Past histories were excluded as constitutional parameters in comparisons of the cases and controls because of unavoidable information bias due to the differences in data collection methods. Missing data were removed from the analyses, and the remaining data were used.

### Ethical consideration

All procedures involving human participants were performed in accordance with the ethical standards of the institutional review board (IRB) of Nagasaki University Hospital (1602221–2) and Nagasaki Renal Center (Nagasaki, Japan) (30001) and of the 1964 Declaration of Helsinki and its later amendments. Although all patients in this study were informed of the investigations being performed, the study was a medical record-based retrospective analysis and the included patients were anonymized. Therefore, the IRB approved the exemption from obtaining written informed consent.

## Results

### Demographic data for patients with cerebral hemorrhage

**Fig. 1:**
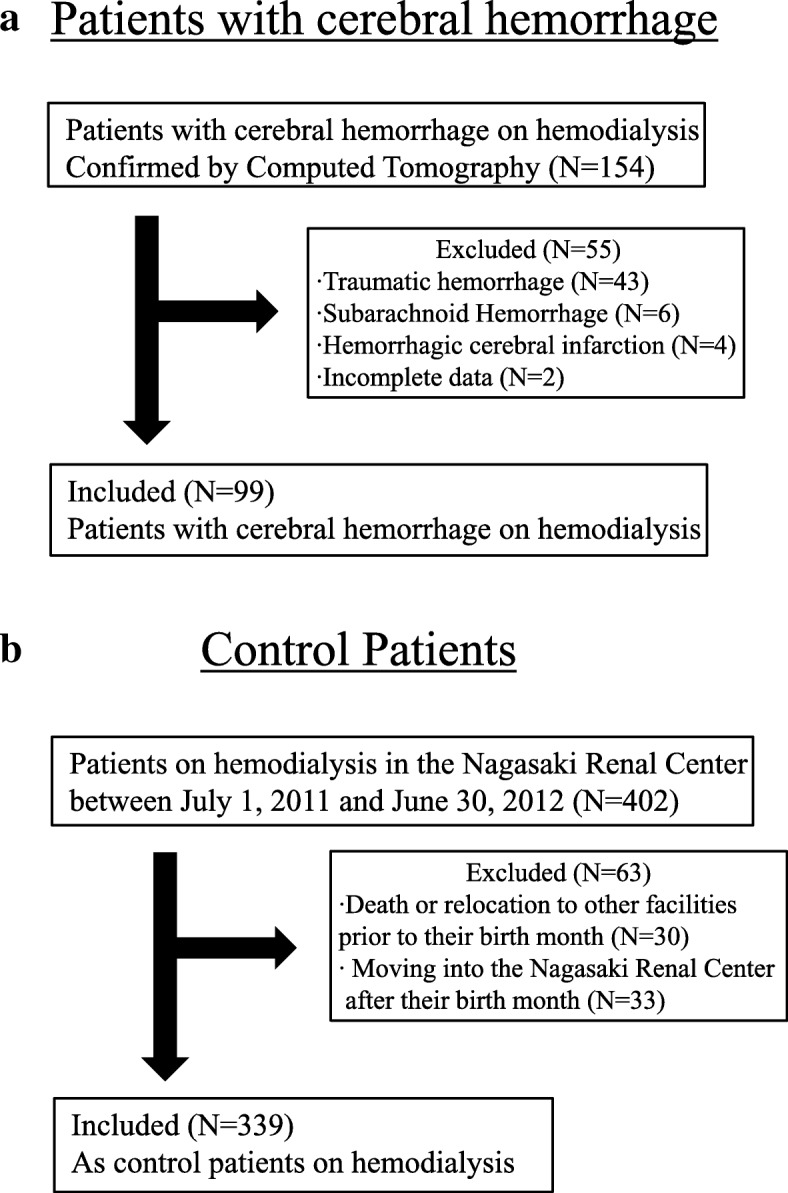
Patient flow diagram. **a** Patients with cerebral hemorrhage in Nagasaki University Hospital. **b** Control patients in the Nagasaki Renal Center between July 1, 2011 and June 2012

**Fig. 2:**
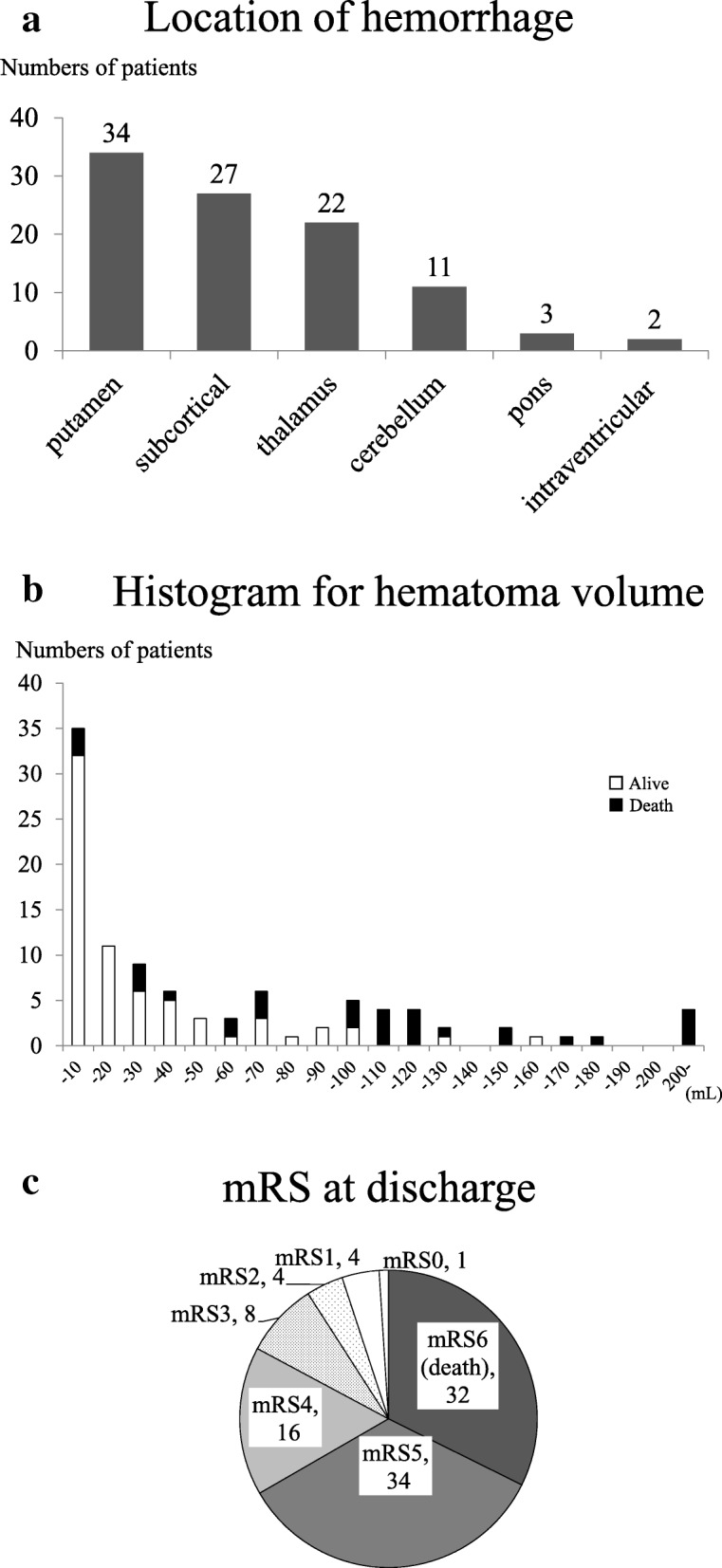
Characteristics of patients with hematoma. **a** Location of hematoma. **b** Distribution of hematoma volume. **c** Neurological severity outcomes, as a severity of cerebral hemorrhage. The numbers in these figures represent the numbers of patients

The participants included 96 HD patients with cerebral hemorrhage who were admitted to Nagasaki University Hospital between 2007 and 2017 and 3 HD patients who developed cerebral hemorrhage in the hospital. The patient flow diagram is shown in Fig. 1. The severity of hemorrhage in terms of GCS, NIHSS, hematoma volume at admission, and mRS at discharge is shown in Table 1. The average age was 64.8 ± 10.7 years (men, 66; women, 33), and the median dialysis vintage was 87 months (Table 2). The median date of the onset of cerebral hemorrhage was May 21, 2012. Figure 2a shows hemorrhagic areas, and Fig. 2b shows the distribution of the hematoma volume. Severe outcomes were observed in 66 patients (67%): 32 died (mRS: 6) and 34 were severely disabled (mRS: 5). Figure 2c shows the total mRS score at discharge.

**Table 1:** Cerebral hemorrhage patients’ condition on admission

	Total (*N* = 99)	Died (*N* = 32)	Survived (*N* = 67)	*P* value
Features of cerebral hemorrhage				
sBP on admission (mmHg)	184 ± 34	187 ± 36	183 ± 33	0.7
dBP on admission (mmHg)	96 ± 23	100 ± 25	94 ± 22	0.3
Pulse pressure on admission (mmHg)	80 ± 18	81 ± 24	80 ± 15	0.9
Glasgow Coma Scale	10 ± 4	6 ± 3	12 ± 4	< 0.001
NIHSS	15 ± 10	23 ± 9	11 ± 9	< 0.001
Intraventricular hemorrhage (%)	64	97	48	< 0.001
Volume of hematoma (mL)	50 ± 60	103 ± 70	24 ± 33	< 0.001
Surgical hematoma evacuation (%)	31	34	30	0.7
Onset during 2-day interval HD (%)	37	41	36	0.6
Time for patients to transport (hour)^a^	4 (3–12)	6 (3–12)	4 (3–11)	0.4

*dBP* diastolic blood pressure, *HD* hemodialysis, *NIHSS* National Institute of Health Stroke Scale, *sBP* systolic blood pressure

Continuous variables are shown as mean ± standard deviation and categorical variables, as percentage or number (percentage)

^a^ Median (interquartile range)

**Table 2:** Demographic data of patients with cerebral hemorrhage divided by dead or alive and control patients

	Died (*N* = 32)	Survived (*N* = 67)	*P* value	CH all (*N* = 99)	Control (*N* = 339)	*P* value
Age (year)	64.9 ± 9.2	64.7 ± 11.5	0.7	64.8 ± 10.7	67.4 ± 13.3	0.06
Male (%)	62.5	68.7	0.5	66.7	57.2	0.09
HD vintage (months)^a^	85 (30–169)	88 (27–177)	0.7	87 (29–177)	56 (23–122)	0.04
Cause of ESRD						
Chronic glomerulonephritis (%)	28	48		41	38	
Diabetes (%)	44	31		35	28	
Nephrosclerosis (%)	13	7		9	17	
ADPKD (%)	6	7		7	4	
Others (%)	6	6		6	6	
Unknown (%)	3	0	0.3	1	7	0.02
Past history						
Diabetes mellitus (%)	44	37	0.5	39	35	0.4
Ischemic heart disease (%)	34	9	0.002	17	34	< 0.001
Atrial fibrillation (%)	3	12	0.11	9	10	0.8
Heart valve repair or replacement (%)	16	2	0.008	6	4	0.2
History of cerebral infarction (%)	31	34	0.8	33	25	0.09
History of cerebral hemorrhage (%)	16	13	0.8	14	6	0.02
Medication						
Number of antihypertensive drugs	1.6 ± 1.2	1.9 ± 1.3	0.3	1.8 ± 1.3	1.6 ± 1.5	0.09
Antiplatelet use (%)	65	36	0.008	45	39	0.3
Warfarin use (%)	25	7	0.02	13	7	0.07
Statin use (%)	17	9	0.3	11	16	0.3
Vitamin D use (%)	54	66	0.3	62	67	0.1
Phosphate binder use (%)	71	68	0.8	69	65	0.5
CaCO_3_ use (%)	52	47	0.7	48	48	1.0
Cinacalcet use (%)	13	18	0.6	16	17	0.9
Walking independently (mRS≦3) (%)	77	86	0.2	83	80	0.5
Dialysis conditions						
Dialyzing time (hr)^a^	4 (3–4)	4 (3.5–4)	0.7	4 (3.5–4)	4 (3–4)	0.02
Dry weight (kg)	52.4 ± 11.5	53.7 ± 11.5	0.4	53.3 ± 11.5	52.1 ± 11.0	0.3
Fluid removal per dry weight proximate to the onset (%)	3.14 ± 2.04	3.35 ± 1.71	0.6	3.29 ± 1.81	3.10 ± 1.74	0.3
Dialysate Ca concentration (mEq/L)	2.81 ± 0.14	2.79 ± 0.18	0.7	2.79 ± 0.17	2.75	NA
Online HDF (%)	13	7	0.4	9	22	0.002
sBP pre-HD proximate to the onset (mmHg)	167 ± 31	167 ± 22	1.0	167 ± 25	150 ± 24	< 0.001
dBP pre-HD proximate to the onset (mmHg)	86 ± 14	87 ± 16	0.8	87 ± 16	78 ± 13	< 0.001
Pulse pressure proximate to the onset (mmHg)	81 ± 24	80 ± 15	1.0	80 ± 18	72 ± 21	< 0.001
ESA (U/week)	6719 ± 3592	6296 ± 4556	0.6	6435 ± 4247	5513 ± 4396	0.03
Epoetin (%)	38	28		31	9	
Darbepoetin (%)	41	37		38	47	
CERA (%)	13	18		16	27	
Intravenous iron use (%)	22	27	0.6	25	19	0.08
Laboratory parameters						
Hb (g/dL)	10.6 ± 1.9	10.6 ± 1.5	0.4	10.6 ± 1.7	10.8 ± 1.4	0.09
Ferritin (ng/mL)^a^	139 (54–284)	71 (31–167)	0.095	85 (34–212)	53 (21–154)	0.03
TSAT (%)	21.3 ± 11.3	24.4 ± 12.9	0.4	23.4 ± 12.4	24.3 ± 13.0	0.6
BUN (mg/dL)	61.4 ± 20.7	60.2 ± 14.9	0.8	60.6 ± 16.8	68.1 ± 18.2	< 0.001
Creatinine (mg/dL)	9.1 ± 3.2	10.0 ± 3.1	0.4	9.8 ± 3.1	10.2 ± 3.4	0.2
Corrected serum calcium (mg/dL)	9.93 ± 0.91	9.27 ± 0.86	0.001	9.48 ± 0.93	9.22 ± 0.92	0.01
Phosphate (mg/dL)	5.47 ± 2.09	4.93 ± 1.47	0.3	5.09 ± 1.68	5.58 ± 1.57	0.007
iPTH (pg/mL)^a^	122 (45–319)	124 (45–202)	0.7	124 (45–202)	72 (28–155)	0.007
ALP (IU/L)	305 ± 103	285 ± 143	0.14	291 ± 131	282 ± 133	0.4
Serum albumin (g/dL)	3.41 ± 0.56	3.62 ± 0.50	0.07	3.55 ± 0.53	3.56 ± 0.43	0.9
CRP (mg/dL)^a^	0.39 (0.09–0.69)	0.17 (0.10–0.61)	0.3	0.20 (0.09–0.65)	0.18 (0.07–0.53)	0.2
T-Chol (mg/dL)	152 ± 35	156 ± 38	0.9	155 ± 37	162 ± 37	0.07
Triglyceride (mg/dL)	102 ± 60	89 ± 44	0.5	93 ± 49	106 ± 64	0.2

*ADPKD* autosomal dominant polycystic kidney disease, *ALP* alkaline phosphatase, *BUN* blood urea nitrogen, *CERA* continuous erythropoietin receptor activator, *CRP* C-reactive protein, *dBP* diastolic blood pressure, *dBP pre-HD* diastolic blood pressure pre-hemodialysis, *ESRD* end-stage renal disease, *HD* hemodialysis, *iPTH* intact parathyroid hormone, *NIHSS* National Institute of Health Stroke Scale, *sBP* systolic blood pressure, *sBP pre-HD* systolic blood pressure pre-hemodialysis, *T-Chol* total cholesterol, *TSAT* transferrin saturation

Continuous variables are shown as mean ± standard deviation and categorical variables, as percentage or number (percentage)

^a^ Median (interquartile range)

Significant differences were observed in the GCS, NIHSS, intraventricular hemorrhage, and volume of hematoma between patients who died and those who survived (Table 1).

### Characteristics of HD patients with cerebral hemorrhage and controls

Patients with cerebral hemorrhage and controls were compared to clarify the correlations for the onset of cerebral hemorrhage. The number of patients who underwent HD in the Nagasaki Renal Center between July 1, 2011, and June 30, 2012, was 402; however, 30 were excluded because of death or relocation to other facilities before their birth month, and 33 were excluded because of moving to our facility after their birth month. Therefore, the number of control patients on HD was 339 (Fig. 1). Table 2 (right side) shows the demographic data of patients with cerebral hemorrhage and the controls.

### Differences between patients who died and those who survived following cerebral hemorrhage

Table 2 (left side) shows patients’ background data, which were confirmed or examined during the pre-critical stage, and the differences between patients who died and those who survived. Significant differences were observed in the past histories of ischemic heart disease and heart valve repair or replacement, use of antiplatelet drugs and warfarin, and corrected serum calcium levels.

According to a multivariate logistic analysis, corrected serum calcium, antiplatelet use, and heart valve repair or replacement were independently associated with the severity of cerebral hemorrhage. The corrected serum calcium had the greatest effect on cerebral hemorrhage among them (*P* < 0.001) (Table 3).

**Table 3:** Univariate and multivariate logistic regression analyses for prognosis

	Univariate			Model 1			Model 2		
	OR	95% CI	*P* value	OR	95% CI	*P* value	OR	95% CI	*P* value
Age per year	1.00	0.96–1.04	0.9	1.00	0.96–1.05	0.9			
Male vs. female	0.76	0.31–1.84	0.5	0.88	0.34–2.32	0.8			
Dialysis vintage	1.00	0.95–1.07	0.8						
History of diabetes	1.33	0.56–3.14	0.5						
Ischemic heart disease	5.33	1.75–16.2	0.002						
Heart valve repair or replacement	12.0	1.34–108	0.03				32.4	2.61–403	0.007
Antiplatelet use	3.26	1.34–7.93	0.008				4.30	1.52–12.2	0.006
Warfarin use	4.13	1.23–13.9	0.02						
sBP pre-HD proximate the onset per 10 mmHg	1.04	0.92–1.18	0.5						
Corrected serum calcium per mg/dL	2.38	1.39–4.09	< 0.001	2.37	1.42–4.23	< 0.001	2.91	1.58–5.35	< 0.001
Phosphate per mg/dL	1.21	0.93–1.58	0.2						
iPTH per 10 pg/mL	1.01	0.97–1.05	0.7						
Serum albumin per g/dL	0.46	0.20–1.05	0.06						

*CI* confidential interval, *OR* odds ratio

Model 1: including age, sex, and corrected serum calcium

Model 2: including repair and/or replacement, antiplatelet use, and corrected serum calcium. Valve repair and/or replacement and antiplatelet use were selected by the stepwise method (mixed method)

### Differences between cerebral hemorrhage group and control group

As shown in Table 2 (right side), significant differences were noted in the dialyzing time, BP pre-HD, proportion of online hemodiafiltration (HDF), erythropoiesis-stimulating agent (ESA) doses, ferritin levels, blood urea nitrogen (BUN) levels, corrected serum calcium levels, serum phosphate levels, and iPTH between cerebral hemorrhage group and control group.

The univariate logistic regression model was applied to these factors and the predetermined traditional risk factors, and multivariable logistic regression analysis was performed subsequently. Model 1 was composed of risk factors for traditional cerebral hemorrhage, and model 2 was created by stepwise methods. Although sBP pre-HD had the greatest association with cerebral hemorrhage in both models, the corrected serum calcium and iPTH were also significant parameters for cerebral hemorrhage (Table 4).

**Table 4:** Univariate and multivariate logistic regression analyses for the onset of cerebral hemorrhage

	Univariate			Model 1			Model 2		
	OR	95% CI	*P* value	OR	95% CI	*P* value	OR	95% CI	*P* value
Age per year	0.98	0.97–1.00	0.08	0.99	0.97–1.02	0.5			
Male vs. female	1.49	0.93–2.39	0.09	1.63	0.86–3.11	0.1			
HD vintage per year	1.02	0.99–1.05	0.14	1.02	0.98–1.07	0.3			
History of diabetes	1.22	0.77–1.95	0.4	1.01	0.51–2.01	0.9			
Number of antihypertensive drugs	1.09	0.93–1.27	0.3						
Warfarin use	1.98	0.97–4.05	0.07				1.45	0.48–4.41	0.5
Dialyzing time per hour	1.54	1.05–2.23	0.03				1.55	0.95–2.52	0.08
Online HDF	0.36	0.17–0.74	0.002				0.42	0.17–1.01	0.052
ESA per 100 U	1.00	1.00–1.01	0.07				1.00	1.00–1.01	0.5
Intravenous Fe	1.61	0.95–2.70	0.07				1.60	0.81–3.22	0.2
sBP pre-HD per 10 mmHg	1.33	1.21–1.47	< 0.001	1.41	1.23–1.61	< 0.001	1.40	1.23–1.59	< 0.001
Hb per g/dL	0.88	0.75–1.04	0.13	0.82	0.66–1.02	0.07			
Ferritin per 10 ng/mL	1.01	1.00–1.02	0.2						
BUN per 10 mg/dL	0.79	0.70–0.90	< 0.001				0.85	0.71–1.01	0.06
Corrected serum calcium per mg/dL	1.40	1.07–1.83	0.01	1.52	1.05–2.20	0.03	1.54	1.07–2.22	0.02
Phosphate per mg/dL	0.82	0.70–0.95	0.007	0.81	0.66–0.98	0.03	0.86	0.71–1.05	0.1
iPTH per 10 pg/mL	1.02	1.01–1.04	0.02	1.03	1.00–1.05	0.03	1.03	1.01–1.05	0.02
T-Chol per 10 mg/dL	0.95	0.89–1.01	0.12						

*BUN* blood urea nitrogen, *CI* confidential interval, *ESA* erythropoiesis-stimulating agent, *HD* hemodialysis, *HDF* hemodiafiltration, *iPTH* intact parathyroid hormone, *OR* odds ratio, *sBP pre-HD* systolic blood pressure pre-hemodialysis

Model 1: Adjusted by age and sex

Model 2: Adjusted by corrected serum calcium, dialyzing time, online hemodiafiltration, sBP pre-HD, BUN, phosphate, iPTH (parameters were selected by the stepwise method (mixed method))

### Predictors of hematoma volume

To determine the predictors and identify the relationship between the corrected serum calcium level and hematoma volume, multiple regression analysis was performed. Model 1 consisted of predetermined factors, and model 2 included use of antiplatelet in addition to the model 1 factors. Multiple regression analysis indicated that the size of the hematoma was significantly correlated with the corrected serum calcium level (β 0.378; standard error (SE) 0.101; P < 0.001) and antiplatelet use (β 0.378; SE 0.089; *P* = 0.005) (model 2; R^2^ = 0.26); however, sBP pre-HD proximate to the onset had no significant correlation with hematoma volume (*P* = 0.09) (Table 5).

**Table 5:** Multiple linear regression analysis model for hematoma volume (logarithmic converted)

	Model 1			Model 2		
	β	Standard Error	*P* value	β	Standard Error	*P* value
Age (year)	0.011	0.008	0.2	0.007	0.008	0.4
Female	−0.031	0.086	0.7	−0.013	0.089	0.9
sBP pre-HD proximate the onset(/mmHg)	0.007	0.003	0.08	0.006	0.003	0.09
Corrected serum calcium (mg/dL)	0.381	0.093	0.02	0.378	0.101	< 0.001
Antiplatelet use				0.24	0.089	0.005
R^2^		0.17			0.26	

*dBP pre-HD* diastolic pressure pre-hemodialysis, *iPTH* intact parathyroid hormone, *sBP pre-HD* systolic blood pressure pre-hemodialysis

Model 1: including age, sex, sBP pre-HD proximate the onset, corrected serum calcium

Model 2: in addition to the model 1’s parameter, antiplatelet use was selected by stepwise method (mixed method)

### Neurological prognosis and corrected serum calcium level

**Fig. 3:**
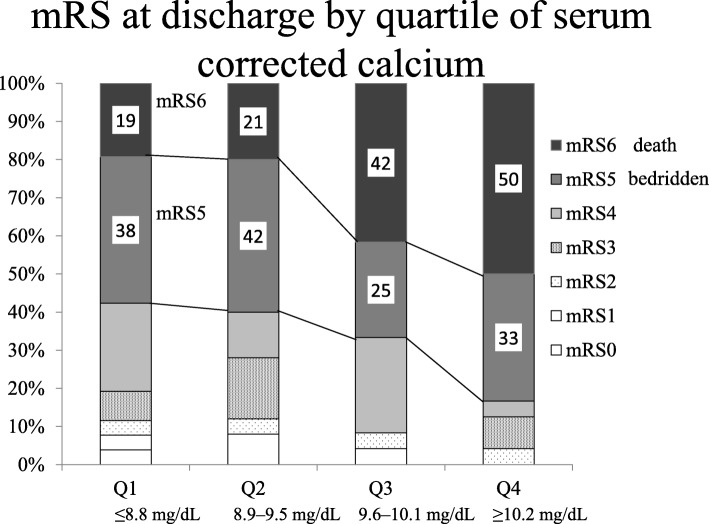
Distribution on the modified Rankin scale score at discharge by the quartile of the corrected serum calcium. Numbers represent the proportions of patients according to the modified Rankin scale. mRS: modified Rankin Scale

To elucidate the corrected serum calcium levels and neurological prognoses of cerebral hemorrhage, the patients with cerebral hemorrhage were divided into four groups according to the corrected serum calcium levels (Q1–Q4): Q1 (corrected serum calcium ≤8.8 mg/dL), Q2 (8.9–9.5 mg/dL), Q3 (9.6–10.1 mg/dL), and Q4 (≥10.2 mg/dL), and the severity of ADL (mRS) was analyzed. The proportions of patients with severe cerebral hemorrhage, who were mRS5 (severe disabled, bed ridden) and mRS 6 (in-hospital death) were as follows: Q1, 57%; Q2, 63%; Q3, 67%; and Q4, 83% (Fig. 3). In addition to patients who died and those who survived, the severity of ADL (mRS) at discharge was also significantly associated with high calcium levels (*P* = 0.02 by Cochran-Armitage test).

### Correlation between serum calcium levels and factors that could affect serum calcium levels in patients with cerebral hemorrhage

We attempted to elucidate factors that affected the serum calcium levels in patients with cerebral hemorrhage as follows: serum phosphate level, iPTH level, serum alkaline phosphatase (ALP) level, vitamin D, cinacalcet, calcium bicarbonate, dialysate calcium level, and before onset ADLs (cut-off point was mRS 3; mRS ≤3 could walk independently), which were shown by the quartiles of the corrected serum levels. However, no significant difference was observed in the serum calcium levels and any of the mentioned factors (Additional file 1 Table 1).

## Discussion

To our knowledge, this is the first study to elucidate the association between serum calcium and cerebral hemorrhage in patients on HD. The corrected serum calcium was positively correlated with the prognosis: disabilities and death from cerebral hemorrhage, hematoma volume, and onset of cerebral hemorrhage in HD patients. After cerebral hemorrhage, more than half of the patients were bedridden or dead (Fig. 3), and the occurrence of cerebral hemorrhage in HD patients should be reduced.

According to previous studies [9, 12], no significant association was found between serum calcium and cerebral hemorrhage; however, data proximate to the cerebral hemorrhage onset were not available in these studies. In our study, serum calcium values were collected close to onset of cerebral hemorrhage and were associated with the prognosis and deterioration of ADL after cerebral hemorrhage (Table 2, Fig. 3). Although precise ADL levels before onset could not be evaluated in the cerebral hemorrhage group, the proportion of walking independently in this group was not very low (Table 2). In this study, the higher the serum calcium level, the lower the ADL after cerebral hemorrhage (Fig. 3).

In the general population, a lower serum calcium level is inversely associated with hematoma volume and poor outcomes [18–20]. In our study, there was no in-hospital death in patients whose corrected serum calcium were < 8.4 mg/dL, suggesting that there was no U-shaped or J-shaped phenomenon between the serum calcium and cerebral hemorrhage in HD patients. Although our analyses could not establish the precise relationship between the serum calcium level and cerebral hemorrhage, several possible mechanisms may explain it. First, a high serum calcium level is associated with fragile arteries due to vascular calcification [28]. Second, changes in calcium homeostasis may affect the vascular tone [29] and function of the blood–brain barrier [30]. Calcium may alter the integrity of blood–brain barrier via several molecular mechanisms and direct interaction of calcium ions with junctional proteins [30]. Unlike the general population, the serum calcium level transition during HD should be considered in these patients [31]. Lowering the calcium concentration in the dialysate is associated with cardiovascular instability and arrhythmias [32]; therefore, the use of low calcium concentration dialysate for calcium reduction was not recommended [33]. From these points of view, calcium concentration transition might be associated with cerebral hemorrhage. Third, the possibility that patients with high serum calcium level had low serum albumin and ADL levels cannot be excluded. We could not adjust the pre-onset ADL levels, which may affect the ADL after cerebral hemorrhage (Fig. 3).

In this study, more than half of patients in the cerebral hemorrhage and control groups were prescribed vitamin D. As vitamin D increases the serum calcium level, it is not advisable to use vitamin D for secondary hyperparathyroidism routinely [16]. Recently, calcimimetics, such as cinacalcet and etelcalcetide, have been widely used for secondary hyperparathyroidism [34], and they can be considered as the first-choice treatment for secondary hyperparathyroidism instead of vitamin D. However, a low serum calcium level is a risk factor for adverse effect, such as sudden death due to arrhythmia, then serum calcium monitoring is needed.

Based on previous reports in the general population, antiplatelet therapy was proven to be associated with a significantly increased risk of cerebral hemorrhage [35], subsequent poor prognosis [36], and early hematoma growth [36]; a strong association was found between antiplatelet therapy and a poor outcome and hematoma volume in cerebral hemorrhage. Therefore, HD patients prescribed antiplatelets should be monitored for occurrence of bleeding.

This study has some limitations. Its generalizability may be limited by its retrospective study design. The study was conducted in Nagasaki area, which might make generalization of the present results to other regions difficult. The number of outcomes and patients were not enough to adjust for confounding factors. Information bias, especially in past histories of complications, could not be excluded. HD patients with cerebral hemorrhage received treatment at several HD centers; therefore, the treatment policies and modalities differed among facilities, and interlaboratory discrepancies in blood examinations cannot be excluded.

This study has several notable strengths. First, the details of patients with cerebral hemorrhage were available. Data on the parameters were collected just before the onset of cerebral hemorrhage, and data on detailed neurological parameters, such as GCS, NIHSS, mRS, and hematoma volume, were obtained after its onset, unlike that in previous studies. Second, the number of cases in this study was higher than those in previous studies [2, 3, 7, 9–11]. The incidence of cerebral hemorrhage was lower than that of other cardiovascular complications in HD patients, so the numbers of cases in previous studies was limited. Third, most HD patients with cerebral hemorrhage in the Nagasaki City medical zone, except cases of sudden death, were supposed to be included during the observation period. The Nagasaki City medical zone, surrounded by sea and hills, is geographically isolated. Nagasaki City and the neighboring towns, Togitsu and Nagayo, are among the medical care zone areas in Nagasaki prefecture, and their population has been around 500,000 in the last 10 years; however, the population of HD patients has increased slightly from 1297 to 1464 between 2007 and 2016 [37]. Unlike other facilities in the Nagasaki medical zone, Nagasaki University Hospital has a stroke hotline and a HD center; therefore, almost all HD patients with cerebral hemorrhage were transferred to this hospital.

## Conclusion

To prevent the onset and progression of cerebral hemorrhage, serum calcium levels in HD patients should be monitored. Further examinations of the direct relationship between high serum calcium concentrations and cerebral hemorrhage in HD patients are warranted.

## Additional file

Additional file 1: **Table S1.** Association between corrected serum calcium levels and other parameters by quartiles of the serum corrected serum calcium level. There was no correlation between serum calcium levels and other parameters of patients’ background. (DOCX 17 kb)

## Data Availability

The datasets used and/or analyzed during the current study are available from the corresponding author on reasonable request.

## Abbreviations

ADL: Activities of daily living
ALP: Alkaline phosphatase
BUN: Blood urea nitrogen
CKD: Chronic kidney disease
ESA: Erythropoiesis-stimulating agent
ESRD: End-stage renal disease
GCS: Glasgow Coma Scale
HD: Hemodialysis
HDF: Hemodiafiltration
iPTH: Intact parathyroid hormone
mRS: Modified Rankin scale
NIHSS: National Institutes of Health Stroke Scale
sBP: Systolic blood pressure
