# Supplementary material for: Association between serum calcium levels and prognosis, hematoma volume, and onset of cerebral hemorrhage in patients undergoing hemodialysis
Source: BMC Nephrol. 2019 Jun 7;20:210. doi: 10.1186/s12882-019-1400-4 (PMC6555959; doi:10.1186/s12882-019-1400-4)
Supplement: Supplementary file 1 — Table S1. Association between corrected serum calcium levels and other parameters by quartiles of the serum corrected serum calcium level. There was no correlation between serum calcium levels and other parameters of patients’ background. (DOCX 17 kb) [file 12882_2019_1400_MOESM1_ESM.docx]

**Table S1. Association between the corrected serum calcium levels and other parameters by quartiles of the serum corrected serum calcium level**

|  | Q1  -8.8  (n=26) | Q2  8.9-9.5  (n=25) | Q3  9.6-10.1  (n=24) | Q4  10.2-  (n=24) | P value  trend |
| --- | --- | --- | --- | --- | --- |
| Vitamin D use (%) | 50 | 67 | 71 | 62 | 0.3 |
| CaCO_3_ use (%) | 52 | 54 | 52 | 16 | 0.2 |
| Cinacalcet use (%) | 12 | 24 | 8 | 22 | 0.7 |
| Walking independently (mRS≦3) (%) | 88 | 92 | 79 | 74 | 0.1 |
| Dialysate Ca concentration (mmol/L) | 2.8 ± 0.2 | 2.8 ± 0.2 | 2.8 ± 0.1 | 2.8 ± 0.2 | 0.7 |
| Phosphate (mg/dL) | 4.8 ± 1.5 | 5.5 ± 1.3 | 5.0 ± 1.8 | 5.1 ± 2.1 | 0.6 |
| iPTH (pg/mL) | 134 ± 152 | 185 ± 164 | 173 ± 121 | 118 ± 138 | 0.5 |
| ALP (IU/L) | 265 ± 107 | 281 ± 160 | 311 ± 142 | 309 ± 113 | 0.5 |

Note: Continuous variables are shown as mean ± standard deviation and categorical variables as percentage or number (percentage)

Abbreviations: ADPKD, autosomal dominant polycystic kidney disease; ALP, alkaline phosphatase; BUN, blood urea nitrogen; CRP, C-reactive protein; dBP pre-HD, diastolic blood pressure pre-HD; iPTH, intact parathyroid hormone; sBP pre-HD, systolic blood pressure pre-HD; T-Chol, total cholesterol; TSAT, transferrin saturation
